# Molecular comparison of Neanderthal and Modern Human adenylosuccinate lyase

**DOI:** 10.1038/s41598-018-36195-5

**Published:** 2018-12-20

**Authors:** Bart Van Laer, Ulrike Kapp, Montserrat Soler-Lopez, Kaja Moczulska, Svante Pääbo, Gordon Leonard, Christoph Mueller-Dieckmann

**Affiliations:** 1 Structural Biology Group, European Synchrotron Radiation Facility, CS 40220, F-38043 Grenoble, France; 2 Max Planck Institute for Evolutionary Anthropology, D-04103 Leipzig, Germany; 3 Present Address: The Francis Crick Institute, London, NW1 1AT United Kingdom

## Abstract

The availability of genomic data from extinct homini such as Neanderthals has caused a revolution in palaeontology allowing the identification of modern human-specific protein substitutions. Currently, little is known as to how these substitutions alter the proteins on a molecular level. Here, we investigate adenylosuccinate lyase, a conserved enzyme involved in purine metabolism for which several substitutions in the modern human protein (hADSL) have been described to affect intelligence and behaviour. During evolution, modern humans acquired a specific substitution (Ala429Val) in ADSL distinguishing it from the ancestral variant present in Neanderthals (nADSL). We show here that despite this conservative substitution being solvent exposed and located distant from the active site, there is a difference in thermal stability, but not enzymology or ligand binding between nADSL and hADSL. Substitutions near residue 429 which do not profoundly affect enzymology were previously reported to cause neurological symptoms in humans. This study also reveals that ADSL undergoes conformational changes during catalysis which, together with the crystal structure of a hitherto undetermined product bound conformation, explains the molecular origin of disease for several modern human ADSL mutants.

## Introduction

Genomic comparisons between modern humans and extinct homini such as Neanderthals recently became possible due to improved methods to extract and sequence ancient DNA samples^1^. These comparisons allow the identification of genetic changes which are specific to modern humans, hence providing insights into the differences distinguishing modern humans from our closest relatives on the tree of Life. Currently, genetic changes in around 90 protein coding regions have been identified as resulting in a stable amino acid substitution in the corresponding protein^2,3^. Despite growing interest in the scientific community, it remains largely unknown which of these modern human specific substitutions are functionally significant and contribute to phenotypical differences between modern humans and Neanderthals. While cellular assays using cells carrying Neanderthal genes are currently being performed and expected to yield exciting results^4^, studies at the molecular level are missing. Such biophysical and biochemical evaluation could reveal how modern human-specific substitutions affect protein structure and function and would serve as a bridge between the ongoing genetic and cellular studies.

This work aims at providing a first step in filling this gap in knowledge by reporting a structural, biochemical and biophysical comparison of modern human and Neanderthal adenylosuccinate lyase (ADSL), an enzyme which acquired a single specific substitution (Ala429Val) during modern human evolution^2,3^. While the published genomes from three Neanderthals and one Denisovan all possess the ancestral Ala429 ADSL variant, all available genetic information from present-day humans exhibit exclusively a Val in this position^5–7^. Of the proteins reported to carry modern human-specific substitutions we selected ADSL because of its medical relevance and its association with intelligence and behaviour. Several missense mutations in the *adsl* gene cause ADSL deficiency, a disease characterised by serious neurological and physiological symptoms such as autism, increased aggression, seizures, microcephaly and muscle wasting^8,9^. ADSL is involved in the *de novo* purine biosynthesis and recycling pathways in which it catalyses the conversion of 5-aminoimidazole-(N-succinylocarboxamide) (SAICAR) to 5-aminoimidazole-4-carboxamide ribotide (AICAR) and the conversion of succinyladenosine monophosphate (SAMP) to adenosine monophosphate (AMP)^10^, respectively. Both reactions are β-eliminations in which fumarate is released. Despite extensive research, the molecular basis on how mutations of the protein cause ADSL deficiency is not always well understood.

Here we describe the first crystal structure of a protein from an extinct hominin species and reveal that, similar to the effect of the ADSL deficiency-causing Arg426His substitution^8,11^, hADSL has a decreased thermal stability compared to nADSL. This difference is not translated into a measurable alteration in the enzymology of the purified protein, but we propose that the Ala429Val substitution, similar to the Arg426His substitution, affects protein-protein interactions in purinosomes. In addition, this study shows that ADSL domain 3 is conformationally flexible and we present the high resolution molecular structure of a previously unknown product-bound conformation of ADSL. This conformational flexibility is associated with the catalytic cycle and results in the insertion of Arg396 into the active site, thus explaining how the Arg396His and Arg396Cys substitutions cause ADSL deficiency in present-day humans^8^.

## Results

### nADSL has an increased thermal stability as compared to hADSL

**Figure 1:**
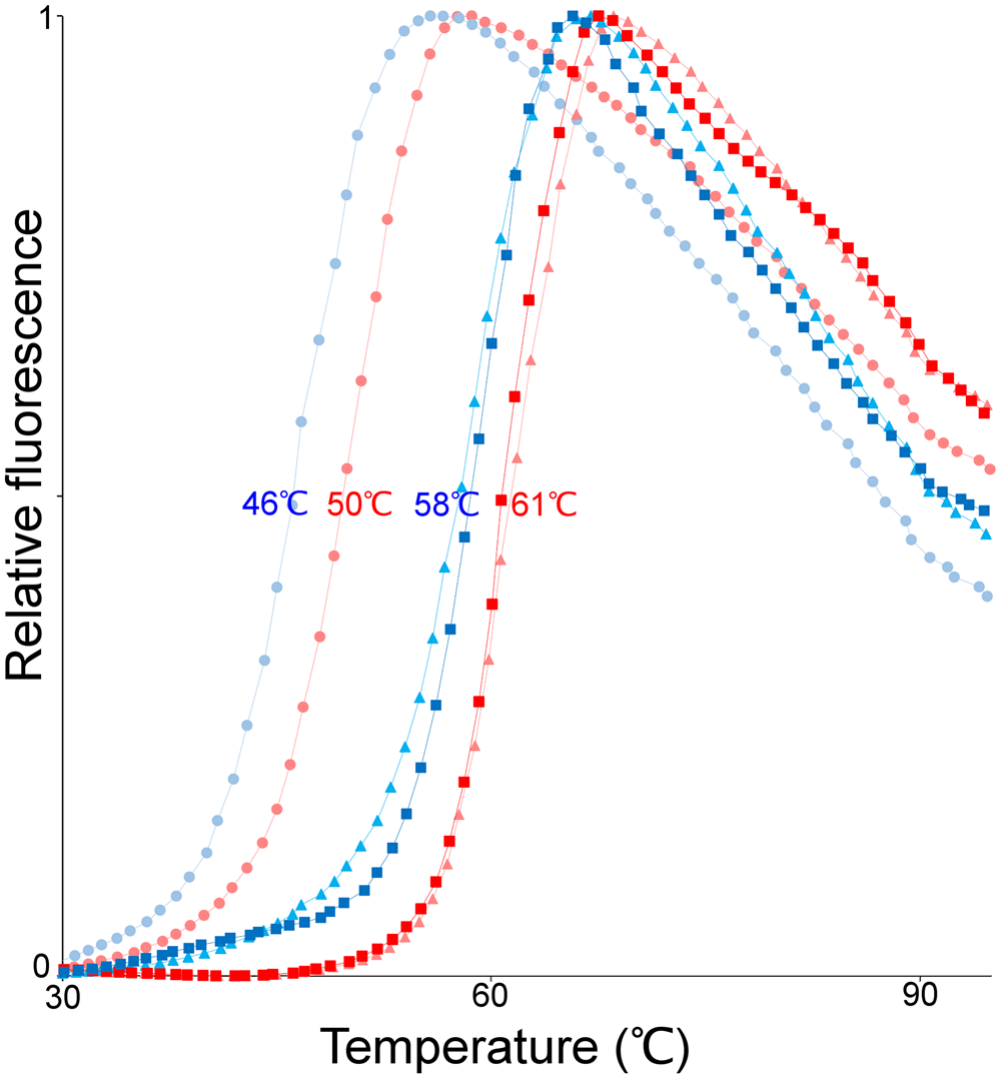
Melting curves of nADSL (red) and hADSL (blue) as determined by a thermal shift assay. The melting curves in three different buffer conditions are shown: in the purification buffer (squares), in water (triangles) and in Bicine buffer at pH 9 (circles). In all three buffers hADSL has a reduced thermal stability compared to nADSL.

Inspection of the crystal structure of hADSL shows that residue 429 is solvent-exposed and located distant from the active site^12,13^. Although one would naturally expect such a substitution not to have large impact on the protein function, some ADSL deficiency-causing substitutions have been reported for solvent exposed residues distant from the ADSL active site. Interestingly, these disease-causing substitutions often affect the protein’s stability^14–16^. In light of this, a comparison of the melting temperatures of modern human and Neanderthal ADSL was carried out. This showed that hADSL has a reduced thermal stability compared to nADSL with melting temperatures of 58 °C and 61 °C for hADSL and nADSL, respectively (Fig. 1). Although this difference in thermal stability is small, it was consistently measured for different preparations of the two proteins and also observed, notwithstanding the clear effect the buffer composition had on the absolute values of the melting temperature (ranging between 50–66 °C for nADSL and 46–63 °C for hADSL), upon varying the ionic strength (between 0 M and 1 M NaCl) and pH (between pH 5 and 10) of the buffer system. Hence, the data reveal that hADSL has a reduced thermal stability in comparison to its Neanderthal homologue.

### Structural comparison of nADSL and hADSL

To investigate possible structural differences between the two proteins, we determined the crystal structures of nADSL in its apo state and bound with the products AMP and fumarate (AMP/fumarate) at resolutions of 1.7 and 2.3 Å, respectively (Table S1) and compared these with the available hADSL structures deposited in the PDB^12,13^.

**Figure 2:**
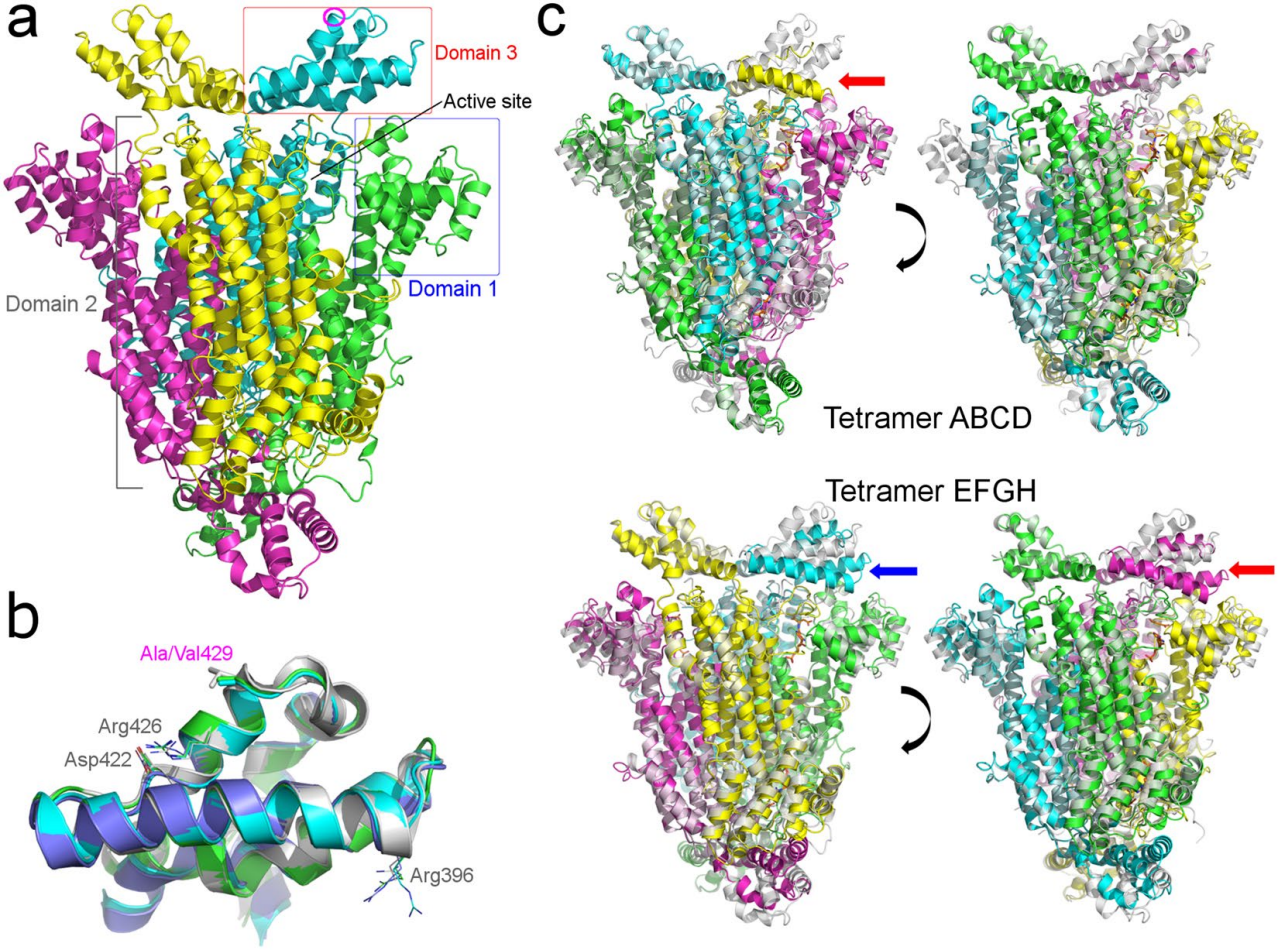
Structural evaluation of nADSL by X-ray crystallography. (**a**) Molecular structure of the apo nADSL tetramer with domain 1 and 3 highlighted by a blue and red box, respectively. The position of the Ala429 residue which distinguishes nADSL from hADSL is indicated in one subunit by a purple circle. (**b**) Superposition of domain 3 from apo hADSL (grey) with nADSL domain 3 in its open (green), semi-closed (cyan) and closed (dark blue) conformation. (**c**) Superposition of apo nADSL (grey) with the two tetramers present in the AICAR/fumarate bound nADSL crystal structure (tetramer ABCD in the top panel and tetramer EFGH in the bottom panel). The active site in which domain 3 is in the closed conformation is marked with a red arrow while the active site in which domain 3 is in the semi-closed conformation is indicated with a blue arrow. Note that domain 3 could not be completely modelled in its closed conformation in tetramer ABCD (top left panel).

The apo structure of nADSL shows an identical tertiary and quaternary structure to that of hADSL, consisting of an all α-helical homotetramer (Fig. 2a) that differs between the two variants with a RMSD (root mean square deviation) between Cα atoms of 0.5 Å. Each protomer within the nADSL and hADSL tetramer can be subdivided into three domains^17^. A central domain 2 (residues 113–364) contains the tetramerisation surface and contributes the catalytic C3-loop (residues 280–300) to the active site^12^. The active site itself is formed by residues from all three domains with each domain originating from a different protomer of the tetramer. Domain 1 (residues 22–112) and domain 2 form the base and one side of the active site thus creating a binding cleft for the substrate, while domain 3 (residues 365–450) resembles a lid which sits on top of the active site (Fig. 2a). It is within this lid domain where the Ala429Val substitution distinguishing nADSL from hADSL is located. This residue is positioned near the end of a first α-helix of a helix-turn-helix motif on the tip of domain 3 and has its side chain solvent exposed. Structurally, domain 3 is the region which displays most of the structural differences between the apo forms of nADSL and hADSL when overlaying the tetrameric structures. However, this difference originates from small rigid body movements (of less than 1 Å) of the complete domain which can also be seen when overlaying the different protomers of nADSL (or hADSL) separately. A superposition of the separate domains 3 of nADSL and hADSL shows that the Ala429Val substitution has no effect on the local structure nor does it influence the local hydrogen bond networks (Fig. 2b).

Comparison of the apo structures of nADSL and hADSL with their respective AMP/fumarate bound ternary complexes reveals few structural changes upon product binding. The most prominent difference can be found in the active sites with some residues involved in product binding being reoriented. Additionally, a small movement of domain 1 and domain 3 towards the active site is observed. Nonetheless, the overall structures are very similar with an RMSD between Cα atoms of 1.0 Å for the hADSL structures and 1.2 Å for the nADSL structures, and, as also seen for the apo structures, no significant structural differences could be detected between the two AMP/fumarate bound structures.

### A new conformation of ADSL

**Figure 3:**
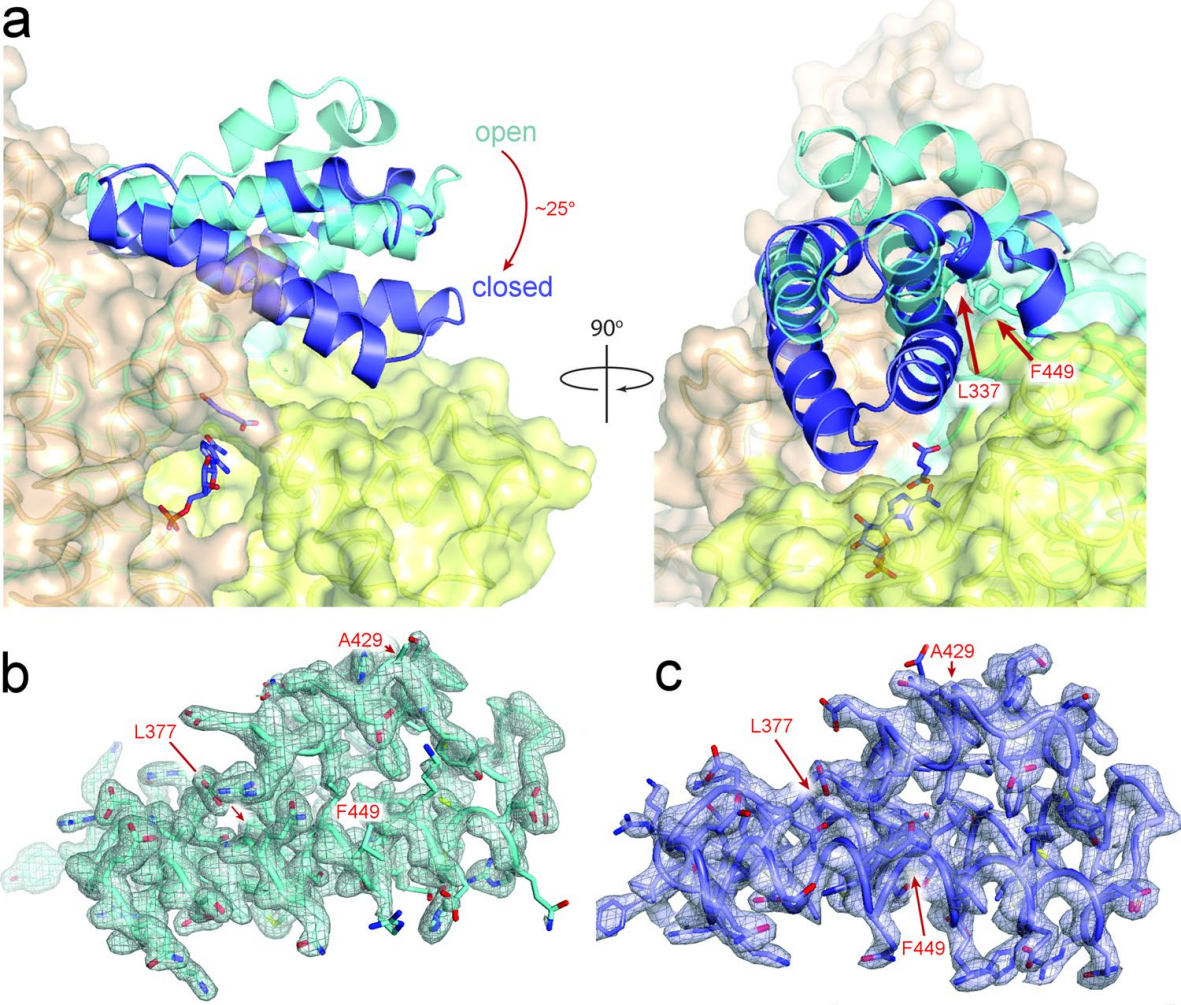
Conformational change of domain 3 in the AICAR/fumarate bound nADSL structure. (**a**) Detail of domain 3 in the open (cyan) and closed (dark blue) conformation. (**b**,**c**) Electron density maps (2F_o_-F_c_) contoured at 1σ level showing domain 3 in the open (**b**) and closed (**c**) conformation. The location of Ala429 and the hinge points for the domain movement (Leu377 and Phe449) are indicated.

Co-crystallisation of nADSL with its other products AICAR and fumarate (AICAR/fumarate) yielded a different crystal form than seen for the apo and AMP/fumarate bound crystals (Table S1). In this new crystal form, two nADSL tetramers are present in the asymmetric unit (tetramer ABCD and tetramer EFGH) in contrast to the single tetramer present in the apo and AMP/fumarate bound structures. Overlay of the different tetramers shows a clear difference in the position of domain 3 between the apo crystal structure and some, but not all, of the protomers of the AICAR/fumarate bound structure (Figs 2c and 3). More specifically, domain 3 is tilted by around 20–25° towards the active site in two protomers of tetramer EFGH (chains G and F) and in one protomer of tetramer ABCD (chain D). As such, it appears that this domain has closed over the active site in these protomers. A closer inspection reveals a small difference in the position of domain 3 between chains G and F with one of them being tilted slightly further towards the active site (chain G). As such, the conformation of domain 3 in the G chain will be referred to as the ‘closed conformation’ in the remainder of the text while the conformation in chain F will be referred to as the ‘semi-closed conformation’. In tetramer ABCD, domain 3 is shifted towards the active site in only one of the protomers (chain D) which, notwithstanding the fact that not all of the electron density is well defined for this domain, mostly resembles the fully closed conformation.

The closed and semi-closed domain 3 conformations have not been previously reported for ADSL and therefore no structure of hADSL in these conformations (nor bound to AICAR/fumarate) is available for comparison. However, since domain 3 closure occurs through a rigid body movement involving two hinge points near its N- and C-terminus (Leu377 and Phe449), the central region of the domain (including residue 429) is structurally identical in all three conformations (Figs 2b and 3). Thus, in case hADSL also adopts the closed and semi-closed conformation no structural difference would be expected between nADSL and hADSL in these conformations.

### ADSL undergoes conformational changes during catalysis

**Figure 4:**
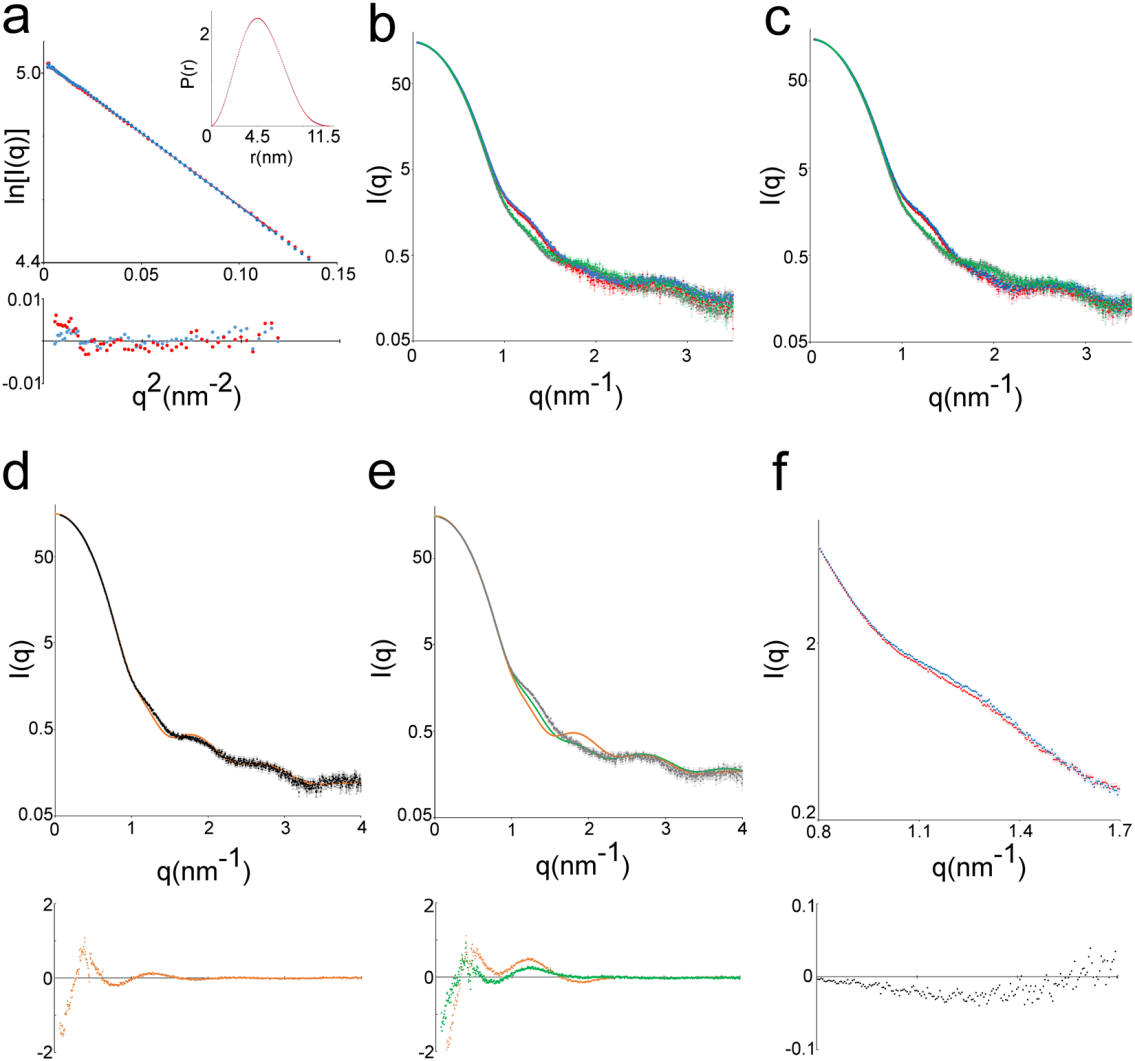
Structural comparison of nADSL and hADSL by small angle X-ray scattering. (**a**) Comparison of the Guinier region and pair distance distribution function (inlet) of apo nADSL (red) and apo hADSL (blue) SAXS data. The residuals to the linear fit of the Guinier function are shown below. (**b**,**c**) Overlay of the SAXS curves from apo (grey), AMP bound (green), AMP/fumarate bound (red) and AICAR/fumarate bound (blue) nADSL (**b**) and hADSL (**c**). (**d**) Overlay of the experimental SAXS curve of apo nADSL (black) with the calculated SAXS curve from the apo nADSL crystal structure (orange). (**e**) Overlay of the experimental SAXS curve of AICAR/fumarate bound nADSL (grey) with the calculated SAXS curve from tetramer EFGH of the AICAR/fumarate bound nADSL crystal structure (green). For comparison, the calculated SAXS curve from the apo nADSL crystal structure is shown in orange. (**f**) Detail of the overlaid 0.8 nm^−1^ < q < 1.7 nm^−1^ region of the scatter curves of AMP/fumarate bound nADSL (red) and hADSL (blue). The residuals to the fit of panel d-f are shown below.

To evaluate whether this conformational change is specific for nADSL or also shared by hADSL, we collected Small Angle X-ray Scattering (SAXS) data from both nADSL and hADSL in their apo and product bound states (Fig. 4 and Table S2). Here, the SAXS curves of the apo state of both proteins clearly deviates from those of the AMP/fumarate and AICAR/fumarate bound proteins indicating that both variants undergo a conformational change upon binding of the products (Fig. 4b,c).

The SAXS curves from apo nADSL and hADSL are identical and fit well the theoretically calculated curves from the corresponding apo crystal structures (for q-values between 0.07 nm^−1^ and 4 nm^−1^; Fig. 4a–d). Hence, consistent with the results obtained by the structural comparison, nADSL and hADSL have an identical in-solution molecular structure in their apo state which is well described by their respective crystal structures. Also the presence of products AMP or AICAR alone does not alter the scattering curves for either nADSL or hADSL (Fig. 4b,c).

In contrast, addition of both AMP/fumarate or AICAR/fumarate to the protein results in a similar altered scattering curve compared to that of the apo proteins (Fig. 4b,c). Hence, both hADSL and nADSL adopt an identical in-solution conformation in the presence of AMP/fumarate and AICAR/fumarate despite the clear difference in the domain 3 orientation between the AMP/fumarate bound and AICAR/fumarate bound nADSL crystal structures. This product bound conformation most closely resembles the conformation of tetramer EFGH of the AICAR/fumarate bound nADSL structure (*i.e*. with two domains 3 in the tetramer closed over their corresponding active sites). This is evident from the theoretically calculated SAXS curve from the nADSL tetramer EFGH structure which better fits (for q-values between 0.07 nm^−1^ and 4 nm^−1^) the experimental SAXS curve than calculated SAXS curves derived from either tetramer ABCD or apo nADSL structures (Fig. 4e). Our SAXS analyses thus show that product binding to ADSL results in the closure of domain 3 as seen in the AICAR/fumarate bound nADSL structure and that the open domain 3 conformation of the AMP/fumarate bound hADSL and nADSL structures reflects a crystallisation artefact (Fig. S1).

The observation that domain 3 is conformationally flexible raises the question whether this conformational change is related to catalysis (and already occurs upon substrate binding) or is associated with product release only. To evaluate this, SAXS data were collected for the inactive nADSL mutant His159Asn^18,19^ in presence of substrate SAMP. The observed scattering curve is identical to that obtained from the AMP/fumarate bound protein showing that domain 3 closes upon substrate binding and remains closed during catalysis (Fig. S2).

Comparison of the scattering curves of nADSL and hADSL reveals a small but reproducible difference (in the 1 to 1.5 nm^−1^ q-range) in the AMP/fumarate (and AICAR/fumarate) bound states (Fig. 4f). Interestingly, this difference is absent in the apo, AMP and AICAR bound SAXS curves. Thus, SAXS analysis reveals a structural difference between nADSL and hADSL that manifests itself only in the product bound state (*i.e*. in the closed and/or semi-closed conformation). Given that the crystal structures of nADSL and hADSL do not reveal any overall structural difference, we propose this difference to be attributed to a change in the dynamics of domain 3 closure.

### Enzyme kinetics and product binding of hADSL and nADSL

**Figure 5:**
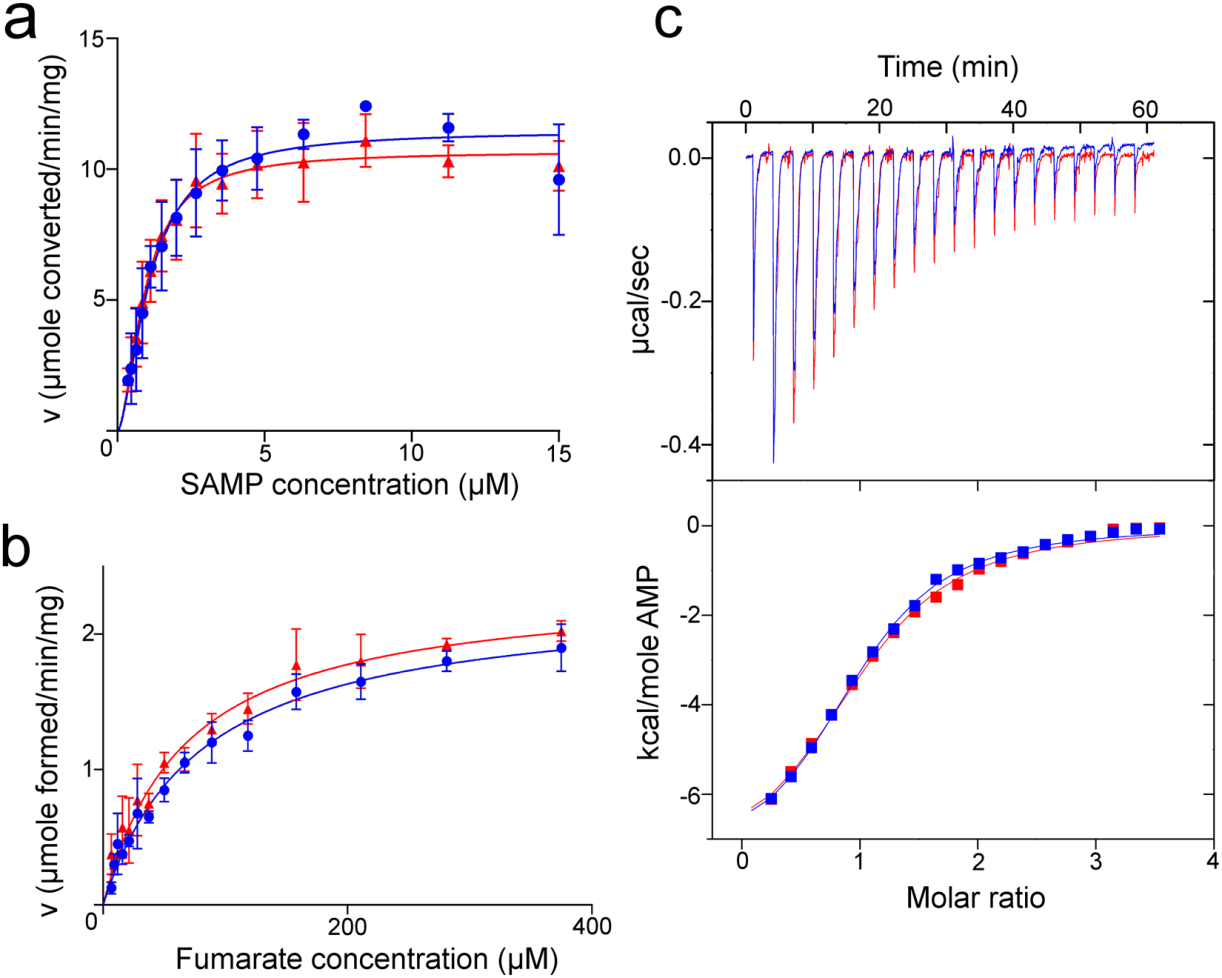
Comparison of enzyme kinetics and product binding by nADSL and hADSL. (**a**) Plots of the specific activities for the forward catalytic reaction of nADSL (red) and hADSL (blue) as a function of the SAMP concentration. (**b**) Plots of the specific activities for the reverse catalytic reaction of nADSL (red) and hADSL (blue) as a function of the fumarate concentration. The mean and standard deviation of three measurements is shown together with the fit to a cooperative enzymatic model (for the forward reaction) or the Michaelis-Menten model (for the reverse reaction). (**c**) Isothermal titration calorimetry assay comparing the binding of AMP to either nADSL (red) or hADSL (blue). Data of a representative experiment are shown (top: calorimetric titration curve, bottom: fitted binding isotherm). A control experiment to determine the heat of dilution is shown in Fig. S3.

To determine whether the Ala429Val substitution has an effect on the enzyme kinetics of ADSL, both the forward (breakdown of SAMP into AMP/fumarate) and reverse (production of SAMP from AMP/fumarate) catalytic reactions of nADSL and hADSL were analysed by monitoring the change in absorbance at λ = 282 nm caused by the conversion of SAMP into AMP. Both proteins display positive cooperativity for the forward reaction with similar kinetic parameters (K_0.5_ of 1.1 µM for both proteins, Hill coefficients of 1.6 and 1.8, and V_max_ of 11.5 and 11.3 µmol min^−1^ mg^−1^ for hADSL and nADSL, respectively; Fig. 5a and Table S3). These values are in agreement with previously reported kinetic data for hADSL^12,14^. For the reverse catalytic reaction no data on the hADSL enzyme have been reported to date. Interestingly, neither hADSL nor nADSL display cooperativity for the reverse direction of the reaction (Fig. 5b and Table S3). Experimental data were fitted using the classical Michaelis-Menten equation, which showed a similar maximum reaction rate for both proteins (2.3 and 2.4 µmol min^−1^ mg^−1^, respectively) and a slightly different Michaelis constant (K_m_ of 80 and 66 µM, respectively) that, nonetheless, remains within the experimental error.

To evaluate if the Ala429Val substitution affects substrate/product binding by modifying the dynamics of domain 3 movement, the binding affinities of both ADSL variants for the product AMP were determined using Isothermal Titration Calorimetry (ITC). These experiments yielded dissociation constants (K_D_) for AMP of around 25 µM for both hADSL and nADSL (Fig. 5c and Table S4) showing that the Ala429Val substitution does not affect product binding.

### Molecular effect of disease-causing substitutions on domain 3

As described above, the Ala429Val substitution is located on domain 3 with its side chain solvent exposed. As such, one would not expect the protein’s enzymology to be affected by the substitution. However, several other substitutions on domain 3 have been reported to cause ADSL deficiency showing that substitutions on this domain can alter the human phenotype^8,9^. To further evaluate this, a comparative analysis of the Ala429Val substitution with three described hADSL deficiency-causing substitutions on domain 3 (Arg396Cys, Asp422Tyr and Arg426His) was performed. Of these substitutions, two (Asp422 and Arg426) are located on the same α-helix as Val429 and also have their side chains solvent exposed. The Arg396 residue on the other hand is located on the opposite side of domain 3 and protrudes into the active site upon domain 3 closure (Fig. 2b).

**Figure 6:**
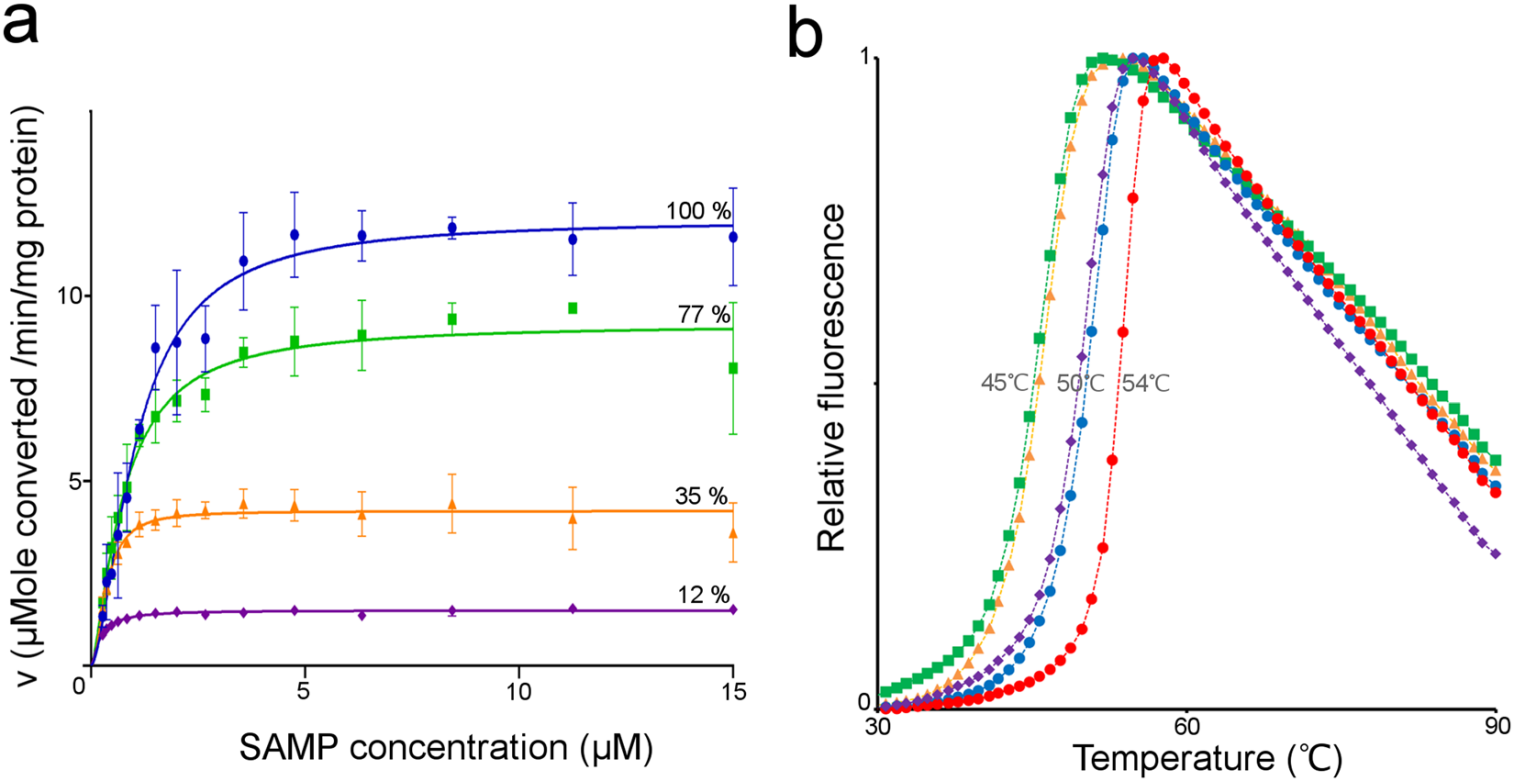
Enzyme kinetics and thermal stability of hADSL disease-causing variants with substitutions in domain 3. (**a**) Plots of the specific activities for the forward catalytic reaction of hADSL (blue circles) and the hADSL variants Arg396Cys (magenta rhombi), Asp422Tyr (orange triangles) and Arg426His (green squares) as a function of the SAMP concentration. The mean and standard deviation of three measurements is shown together with the fit to a cooperative enzymatic model. (**b**) Melting curves of nADSL (red circles), hADSL (blue circles) and the hADSL variants Arg396Cys (magenta rhombi), Asp422Tyr (orange triangles) and Arg426His (green squares) as determined by a thermal shift assay.

In agreement with previous studies, substitution of the Arg396 residue causes a drastic decrease in the enzymatic activity and abolishes the enzyme’s cooperativity (Fig. 6a and Table S5)^11,14^. The loss in activity is not caused by structural instability of the protein as thermal shift assays show that the Arg396His mutant is as stable as native hADSL (Fig. 6b). This residue was previously proposed to be implicated in substrate channelling based on its location^11,14^. However, given that Arg396 is inserted in the active site upon domain 3 closure it is more likely that this residue has a direct role in catalysis.

The Asp422Tyr substitution has a large impact on both the enzymatic activity and thermal stability (Fig. 6 and Table S5). With this residue located far from the active site and not participating in substrate/product binding nor the catalytic reaction itself, the decrease in enzymatic activity of this mutant can be directly related to its reduced stability.

Finally, the Arg426His substitution shows a decreased thermal stability of 5 °C, which is comparable to the effect of the Asp422Tyr substitution. However, the Arg426His substitution retains almost 80% of the native hADSL activity, more than twice that of the Asp422Tyr variant (Fig. 6 and Table S5). This is reminiscent of the behaviour observed for the Ala429Val substitution: a decreased thermal stability without a large impact on the enzymatic activity. Since the Arg426His substitution causes the severe form of ADSL deficiency despite its limited effect on activity^8^ this implies both that the impact of an ADSL substitution on the human phenotype cannot be directly assumed from its effect on the enzyme’s activity and that surface exposed residues on domain 3 can have a functional role which is not directly related to enzymatic activity.

## Discussion

**Figure 7:**
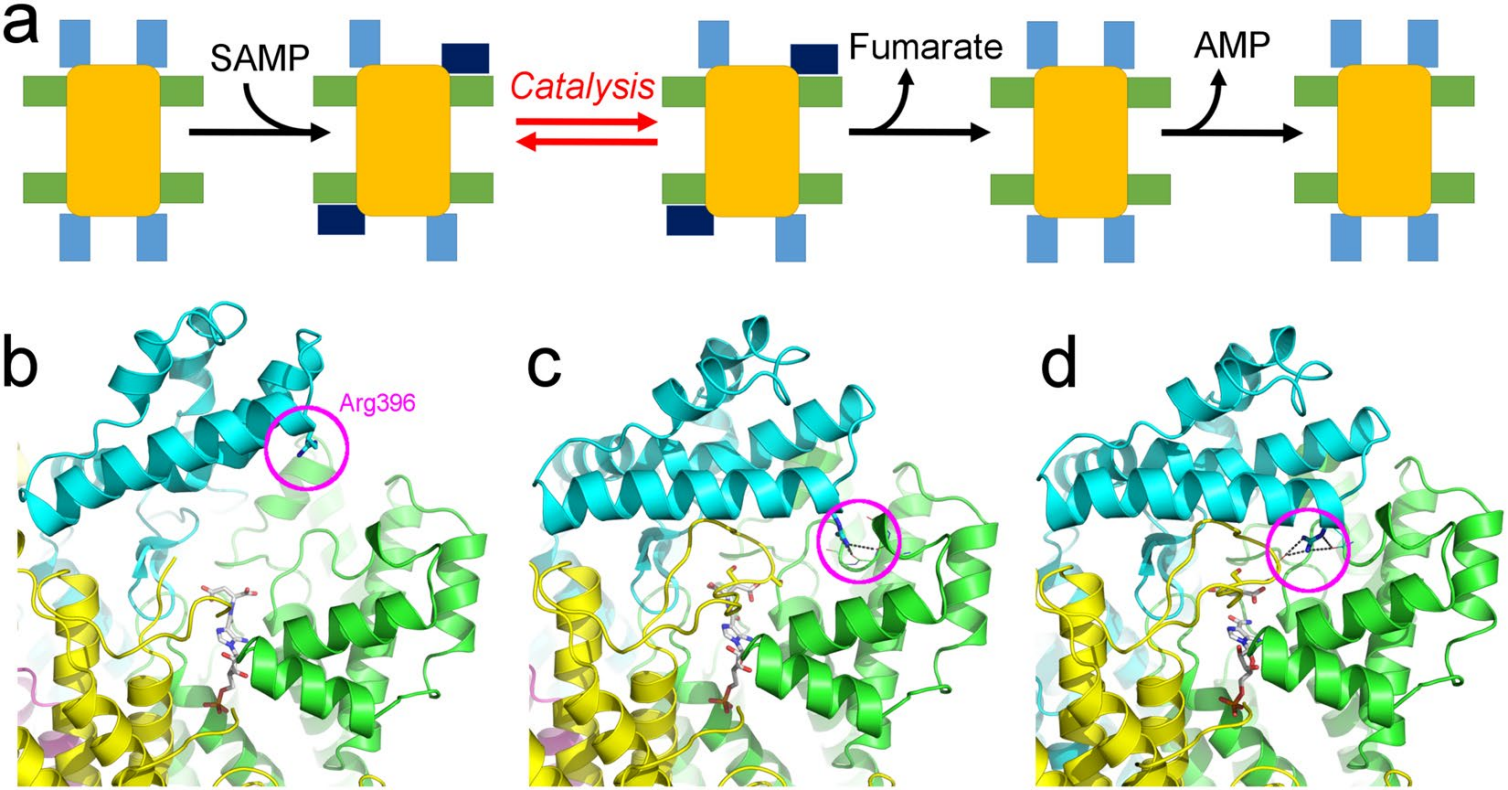
Schematic representation of the conformational changes ADSL undergoes during catalysis (**a**). The domain 2 of the ADSL tetramer is represented as a yellow box while domain 1 and 3 are shown as green and blue boxes, respectively. In the open conformation domain 3 is coloured in light blue, while the closed and semi-closed conformations are coloured in dark blue. Comparison of the location of Arg396 in the open (**b**), semi-closed (**c**) and closed (**d**) domain 3 conformation as seen in the AICAR/fumarate bound nADSL structure. The Arg396 residue is highlighted by a purple circle and the H-bonds in which it is implicated are shown as grey dotted lines. The protein is shown in cartoon representation with domain 1 coloured in green, domain 2 in yellow and domain 3 in blue. The substrate SAICAR (in the open and semi-closed conformation) and products AICAR/fumarate (in the closed conformation) are shown as grey sticks. Note that in the open domain 3 conformation the C3-loop could not be modelled.

Human adenylosuccinate lyase is a well-studied protein for which several mutations have been described to cause ADSL deficiency, a disease characterised by profound psychomotor retardation and currently without an effective therapy^8,9^. Even though crystal structures of hADSL are available^12,13^, questions related to its molecular mechanism and how certain mutations cause disease have remained open. Based on the high structural similarity between the apo and product bound hADSL structures, no large structural movements were expected during catalysis. However, our work now shows that hADSL does undergo conformational changes upon substrate (SAMP or SACAR) binding in the form of a closure of domain 3 over the active site. Following catalysis and release of the product fumarate, ADSL returns to its open conformation allowing the dissociation of the products AMP or AICAR and thus freeing the active site for another round of catalysis (Fig. 7a). This organized opening of domain 3 only after dissociation of fumarate explains the preferential sequential product release observed in ADSL, which releases fumarate prior to AMP^20^. In this scheme, AMP is only able to exit the active site after domain 3 has returned to its open conformation. The linkage between product binding and domain 3 movement implies communication between the active site and domain 3. However, a comparison of the crystal structures presented here does not show a readily identifiable network between the two regions. In fact, the conformation of all residues linking them, apart from those interacting directly with the ligands, remain unaltered in the open, semi-closed and closed conformations. A dynamic equilibrium between the different domain 3 conformations in solution can however be expected based on the high atomic B-factors of the domain. Here, the open conformation would be favoured in absence of products (or substrates), while the closed conformation is favoured in the product bound state. Product binding causes domain 1 to move closer towards the active site due to direct interactions with both AMP and fumarate. In this orientation, the enthalpy gain of interacting with domain 3 could overcome the entropy cost associated with locking domain 3 in the semi-closed or closed conformation. Correspondingly, dissociation of fumarate from the active site and consequential loss of its interactions with domain 1 would push the equilibrium back to the entropically preferred open domain 3 conformation.

**Figure 8:**
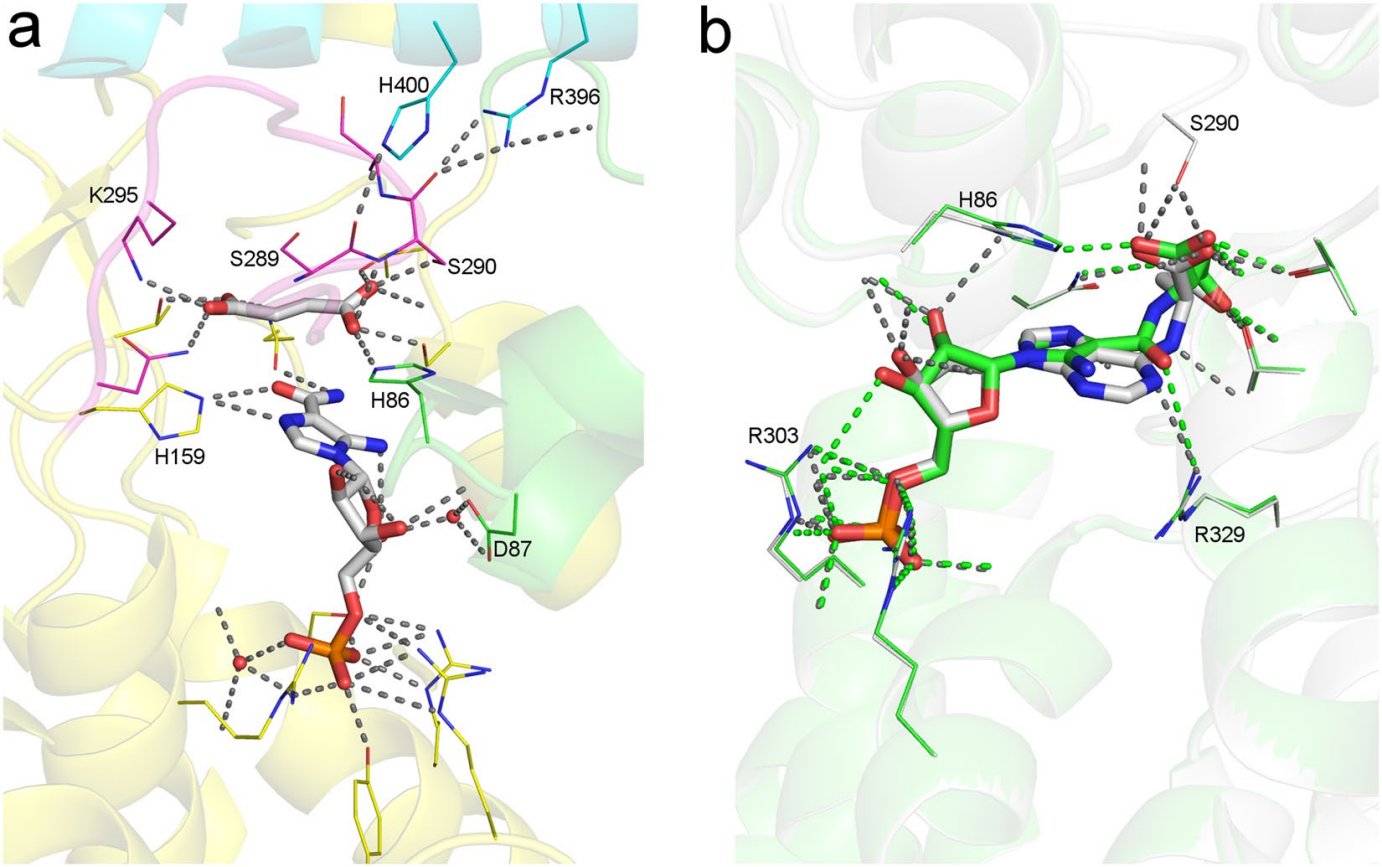
Detail of active site of ADSL. (**a**) Active site of AMP/fumarate bound nADSL with domain 1 in green, domain 2 in yellow, domain 3 in cyan and the catalytic C3-loop in pink. Residues interacting with the products are shown as ball-and-stick and hydrogen bounds as dashed grey lines. (**b**) Overlay of SAICAR (green) and SAMP (grey) bound in the active sites of nADSL. Interacting residues are shown as ball-and-stick, hydrogen bonds with SAICAR as dashed green lines and hydrogen bonds with SAMP as grey dashed lines.

The AICAR/fumarate bound nADSL structure detailed here represents the first high-resolution model of a hominin ADSL protein with domain 3 in a semi-closed or closed conformation. This structure clearly explains why the Arg396His and Arg396Cys mutations abolish enzymatic cooperativity and cause disease in humans. In the hADSL crystal structures, with all domains 3 in the open conformation, Arg396 is located on the tip of domain 3 distant from the active site and was therefore reported to be involved in substrate channelling^9,11,14^. However, in the AICAR/fumarate bound nADSL structure, Arg396 is inserted into the active site and forms part of a hydrogen bond network involving residues from domain 1 in the semi-closed conformation and the catalytic C3-loop (belonging to domain 2) in the fully closed conformation (Fig. 7b–d). We propose that these interactions are at the origin of the communication between the different active sites in the ADSL tetramer, thus permitting cooperativity. The formation of the H-bond networks would be impaired by the ADSL deficiency-causing Arg396His and Arg396Cys substitutions, thus explaining why these substitutions destroy cooperativity. In addition, domain 3 closure directly contributes to the enzymatic reaction by structuring the catalytic C3-loop though an H-bond network involving domain 3 residues Arg396, His400 and Arg404. Indeed, electron density for the C3-loop is only clearly defined in the protomers of the AICAR/fumarate bound nADSL structure where domain 3 adopts the closed or semi-closed conformation (Figs 7 and 8a). In contrast, hADSL and nADSL crystal structures in which domain 3 is in the open conformation show ill-defined electron density caused by a highly flexible C3-loop^12,13^. It is interesting to note that in the AICAR/ fumarate bound crystal structure, the products AICAR and fumarate are only found in the active sites in which domain 3 is locked in its closed conformation. In those active sites in which the domain adopts the semi-closed or open conformation, the reverse enzymatic reaction appeared to have occurred in the crystals since they all have substrate SAICAR bound (Fig. S4). Taken together, these results show that domain 3 movement is closely associated with the ADSL enzymology and likely the main actor in regulating the enzymatic cooperativity. Therefore, the human disease-causing substitutions Arg374Trp, Ser447Pro, Thr450Ser and Arg452Pro, which are all located near the hinge points (Leu377 and Phe449), can be explained by affecting domain 3 movement.

The presence of a structured catalytic C3-loop in the crystal structure of AICAR/fumarate bound nADSL allows for the evaluation of the active site in a conformation primed for catalysis (Fig. 8a). ADSL was proposed to utilise a general acid-base mechanism for catalysis based on structural data and kinetic studies^18,21^. Ser289 was identified as the catalytic base which initiates the reaction by abstracting a proton from the succinyl moiety of the substrate. His159 would then act as catalytic acid which protonates the substrate at either the N1 or N6 position facilitating bond cleavage. In contrast to His159, which is optimally positioned to function as the catalytic acid, the location of Ser289 in the AICAR/fumarate bound nADSL structure raises questions on its role as catalytic base. Not only has the residue no direct interaction with the fumarate moiety, its sidechain is orientated away for the carboxyl groups of fumarate at a distance of more than 3.5 Å. In contrast, the adjacent Ser290 residue directly H-bonds with the carboxyl group of fumarate thus being more favourable situated to act as catalytic base. The Ser290 residue would nonetheless need to be primed to be able to abstract a proton from the substrate. This role could be performed by Arg396 and/or His400 since in the closed domain 3 conformation these domain 3 residues H-bonds with the carboxyl backbones of Ser290 and Ser289 (Fig. 8a). As such these H-bonds would not only serve to structure the catalytic C3-loop but also lower the pKa of Ser290 (or Ser289) allowing it to act as catalytic base.

ADSL was shown to be more efficient in the binding and catalytic breakdown of SAICAR than SAMP. An overlay of SAMP and SAICAR bound active sites of nADSL shows that the location of both substrates in the active site is largely identical (Fig. 8b). Ray *et al*. suggested, based on isothermal titration calorimetry assays, this difference in affinity to originate from an addition H-bond between SAICAR and the protein^12^. The structures however show no additional H-bonds between the protein and SAICAR. The difference in affinity between both substrates can be caused by the less stressed binding of SAICAR. Indeed the position of the bond between the succinyl and AMP/AICAR moieties is different between SAICAR and SAMP due to the planarity of the adenine ring of SAMP (Fig. 8b). The difference in catalytic activity for both substrates was previously suggested to originate from a difference in utilised reaction mechanism^12^. SAICAR, in contrast to SAMP, can delocalise the negative charged originating from the deprotonation of the succinyl moiety to its carbonyl O6 atom^12^. Here, Arg329 would be well positioned to stabilise the negative charge, hence lowering the activation energy for the reaction (Fig. 8b). Since SAMP does not possess a carbonyl group at this position, a similar stabilisation of the transition state by Arg329 is impossible. Interestingly, the disease-causing Arg303Cys substitution disproportionately disturbs the breakdown of SAMP compared to SAICAR^11,12^. This residue interacts with the phosphate group of the substrates and is located distant from the site distinguishing SAICAR from SAMP. Together with the identical interaction of this arginine residue with SAMP and SAICAR a direct influence on catalysis seems unlikely (Fig. 8b). This observation thus supports the hypothesis that the difference in catalytic efficiency is caused by the combined effect of a lower binding affinity for the substrates with a more efficient SAICAR catalysis^12^. Here, most substrates would rapidly dissociate from the active site of the Arg303Cys variant after binding due to the loss of interaction of the arginine residue with the phosphate group of the substrates. Since the breakdown of SAICAR is more efficient than that of SAMP, this rapid binding equilibrium would disproportionately impact the catalysis of SAMP compared to SAICAR.

Adenylosuccinate lyase is one of few proteins known to have acquired a fixed substitution in modern humans during homini evolution^2^. The study presented here was initially aimed at determining whether this Ala429Val substitution functionally affects ADSL. Even though we show that enzyme kinetics and product binding are unchanged, this does not exclude that the Ala429Val substitution affects purine metabolism in a cellular environment. In human cells grown under purine limited conditions, ADSL forms part of a transient protein complex called the purinosome which includes all six enzymes of the *de novo* purine biosynthesis pathway and whose formation is suggested to increase the purine biosynthesis rate by improved substrate channelling^22,23^. In this tightly organized arrangement, even subtle variations in individual proteins could affect the purine biosynthesis as a whole. Indeed, ADSL deficiency-causing mutations which decrease hADSL stability, like Arg426His, were shown to negatively impact the purinosome assembly^24^. This impaired purinosome assembly is likely the main contributor to the severe phenotypical effect the Arg426His substitution has on humans considering its rather limited effect on the enzymatic activity of the purified protein alone (Fig. 6). The latter also clearly indicates that the impact of an ADSL substitution on the human phenotype cannot be directly correlated from its effect on the enzymatic activity. Considering the Arg426His substitution affects purinosome assembly it can be speculated that this region of ADSL is involved in protein-protein interactions in the purinosome and that other substitutions in the region, like Ala429Val, can similarly affect purinosome assembly. Unfortunately however, our current knowledge on the purinosome is limited and both the exact nature of ADSL binding partners as well as their binding surface on the ADSL tetramer remain to be determined. It is however noteworthy that certain *Archaea* lack both the helix-turn-helix motif on which Arg426 and Val429 is located in their ADSL homologue^25^ and the downstream protein in the *de novo* purine biosynthesis pathway, 5-aminoimidazole-4-carboxamide ribonucleotide formyltransferase (ATIC)^26^, suggesting that the former helix-turn-helix motif is the binding site for ATIC. A role for this helix-turn-helix motif as recognition element for binding partners^25^ and a direct interaction between ADSL and ATIC has already previously been proposed^27,28^. Thus, taking together with previous work, our data hints towards an effect of the Ala429Val substitution on purinosome assembly and suggests the substitution could affect the human phenotype. Additional studies, in particular cellular or mice assays, are required to validate this hypothesis.

Although the Arg426His and Ala429Val substitution both affect ADSL stability, the molecular origin of this effect is different. The side chain of Arg426His forms H-bonds with residues from the second helix of the helix-turn-helix motif, thus directly stabilising the motif. The molecular origin of the reduced thermal stability of the Ala429Val substitution is less obvious, but can be caused by differences in the hydration shell around domain 3 or by an altered entropy of unfolding. Recently, a difference in the thermal stability of adenylate kinase by glycine substitutions of surface exposed alanine and valine residues located distant from the active site was observed^29^. These substitutions were suggested to entropically affect the dynamics of local unfolding of the domain they are located on and were shown to impact the enzymatic parameters. Therefore, glycine substitutions of surface exposed residues distant from the active site were suggested to be used to fine-tune the enzymatic parameters of protein homologues^29,30^. Interestingly, these substitutions are commonly found in regions or domains whose mobility is necessary for catalysis. In this, it reflects the ADSL Ala429Val substitution. An effect of the Ala429Val substitution on the dynamics of domain 3 would also be consistent with the observed difference in SAXS curve between the product (AMP/fumarate) bound proteins.

The biophysical and biochemical analysis presented here of an enzyme that differ between modern humans and extinct homini fits well in a synergetic approach, in combination with genetic data and *in vivo/in cellulo* studies, to study the impact of these proteins on the human evolution. The current study shows that genetic data and predictions alone are not always sufficient to evaluate the effect of a substitution. Here, a conservative Ala/Val substitution of a solvent exposed residue distant from the active site was investigated. No large effect would be expected from such a substitution, yet our experimental results reveal an altered thermal stability. The hypothesis that this substitution, like the nearby disease-causing Arg426His substitution, can affect the human phenotype through its involvement in purinosome assembly can act as a starting point to design cellular studies. Hence, a multi-disciplinary approach can aid in evaluating the effect protein substitutions have on the human phenotype and hominin evolution.

## Methods

### Expression and purification of ADSL variants

All ADSL variants were expressed with an N-terminal thrombin cleavable histidine tag from a pET-14b vector. The expression and purification protocol was adapted from the protocol described in Lee & Colman, 2006^31^. In short, the ADSL gene containing pET-14b vector was transformed in *E. coli* Rosetta2 (DE3) cells which were grown at 37 °C in Luria Broth media till an optical density at λ = 600 nm of 0.6 was reached. At this point the temperature was reduced to 20 °C and protein overexpression was induced by addition of 1 mM isopropyl β-D-1-thiogalactopyranoside (IPTG). Following overnight overexpression, the cells were harvested by centrifugation and resuspended in buffer A (10 mM sodium phosphate pH 7.5, 500 mM NaCl, 5% glycerol, 5 mM imidazole, 10 mM MgCl_2_, 10 mM ß-mercaptoethanol) supplemented with protease inhibitors, DNase I and lysozyme. The bacterial cells were lysed by sonication and the lysate cleared by centrifugation. Subsequently, the histidine tagged ADSL protein was purified from the lysate by immobilised metal affinity chromatography using a 5 ml GE Healthcare HisTrap HP column. Following a wash step using buffer A supplemented with 50 mM imidazole, elution of the ADSL protein from the column was accomplished by running a gradient towards buffer A supplemented with 500 mM imidazole. Fractions containing pure ADSL (as determined by SDS-PAGE) were pooled and thrombin at 10 units/mg ADSL was added to the mixture in order to remove the N-terminal Histidine tag. The sample was dialysed overnight against 10 mM HEPES pH 7.5, 100 mM NaCl, 1 mM dithiothreitol (DTT) and 2 mM CaCl_2_. After overnight thrombine cleavage, the thrombin and uncleaved ADSL proteins were removed by Histrap HP and benzamidine-sepharose (GE Healthcare) affinity columns. The flow-through of these columns was concentrated and applied on a Superdex 200 (GE Healthcare) size exclusion chromatography column run in 10 mM HEPES pH 7.5, 100 mM NaCl, 1 mM DTT. ADSL eluted as a single peak at an elution volume corresponding to the molecular weight of an ADSL tetramer. Fractions corresponding to this peak were pooled, concentrated and stored at −20 °C.

Due to the appearance of precipitation during the thrombin cleavage of the hADSL mutant D422Y, the thermal shift assay and enzymatic assay (Fig. 6 and Table S5) of the domain 3 mutants (and the control native nADSL and hADSL proteins) were performed with samples not treated with thrombin. Comparison of the obtained data from native nADSL and hADSL with the data obtained from thrombin treated samples showed that the presence of the Histidine-tag did not influence the stability nor enzymology of the proteins.

### Protein stability assays

The thermal shift assays were performed in triplicate according to standard protocols^32^. In short, a two microliter protein solution at 5 mg/mL was mixed with 1 µL of 100x Sypro Orange (Molecular Probes) and 22 µL of buffer in a 96-well PCR plate. The samples were heated from 25 °C till 95 °C while monitoring the fluorescence changes (excitation wavelength at 490 nm and emission wavelength at 575 nm) on a charge-coupled device camera.

### Crystallisation of nADSL and data-collection

High throughput crystallisation screens were set up with nADSL at concentrations of 5, 10 and 20 mg/mL^33^. Small crystals were obtained in several conditions. Manual reproduction of these crystals by the hanging drop vapour diffusion method yielded suitable crystals for X-ray diffraction experiments after 1–2 days at 20 °C. The crystals used for data collection were obtained by mixing 1 µL of nADSL protein solution at 10 mg/mL with 1 µL of a 20% PEG6000, 0.1 M Tris pH 8 crystallisation solution. The obtained crystals were fished in a crystallisation solution supplemented with 25% glycerol prior to flash freezing them in liquid nitrogen.

Crystals of product bound nADSL were obtained from high throughput crystallisation screens at 4 °C using a 10 mg/mL nADSL sample supplemented with 2 mM fumarate and either 2 mM AMP or 2 mM AICAR. Needle shaped nADSL crystals containing AMP and fumarate appeared after 3 days in a drop containing 25% PEG1000, 0.1 M MES pH 6.5 as crystallisation solution. These crystals were harvested and cryo-cooled using the EMBL CrystalDirect Harvester^34^. Initial nADSL crystals containing AICAR and fumarate were obtained in the Morpheus screen (Molecular Dimensions) after 3 days at 4 °C. The crystallisation condition consisted of 20% ethylene glycol, 10% PEG8000, 0.1 M imidazole/MES pH 6.5 and 0.12 M alcohols. Larger needle like crystals were obtained by reproducing the latter crystallisation condition in a hanging drop vapour diffusion experiment mixing 1 µL of protein sample with 1 µL of crystallisation solution. These crystals were harvested and flash frozen in liquid nitrogen.

All X-ray diffraction data were collected at 100 K at the European Synchrotron Radiation Facility (ESRF) in Grenoble, France. Beam line ID29^35^ was used for the apo nADSL data set, while the datasets from the product (AMP/fumarate and AICAR/fumarate) containing nADSL crystals were collected at ID30A-3^36^. Selection of the best diffracting crystal, determination of the optimal data collection strategy and initial data processing was automatically performed by EDNA/BEST in the mxCuBE pipeline^37–40^. A helical data collection strategy was applied for the needle shaped AICAR/fumarate containing nADSL crystal.

### X-ray crystallography data processing, refinement and structure analysis

Data indexing, integration and scaling were performed using the XDS suite^41^ with the data being truncated at high resolution when the <I/σ(I)> value fell below 1 or the CC_1/2_ value below 30%. Initial data quality was assessed by phenix.xtriage^42^. The published structure of tetrameric hADSL^13^ was used as search model to determine the structure of apo nADSL by molecular replacement using Phaser^43^ in the CCP4 software package^44^. This structure was subsequently used as search model for the AMP/fumarate and AICAR/fumarate containing nADSL data sets. Manual and automated model building and refinement cycles were alternated using respectively coot^45^ and Refmac5^46^ and let to the final models in which all temperature factors were isotropically refined. All models were validated using the MolProbity server^47^. Data collection, processing and validation statistics are summarized in Supplementary Table S1. All figures of protein structures were created using Pymol^48^.

### Small angle X-ray scattering data analysis

All SAXS data were collected in batch at 20 °C on beam line BM29 of the European Synchrotron Radiation Facility (ESRF) using the sample changer robot and PILATUS 1 M detector (Dectris) at a distance of 2.864 m^49,50^. The X-ray beam energy was 12.5 keV and the beam size 700 × 700 *μ*m^2^. Ten frames with an exposure time of each one second were collected, normalized to absolute values and averaged starting from a 50 µL sample at 5 mg/mL. Initial data quality assessment, data reduction and buffer subtraction was performed by BioSAXS EDNA and monitored using BsxCuBE and ISPyB^51,52^. In this set-up no signs of radiation damage were detected. Varying the ADSL concentration (between 10 mg/mL and 1 mg/mL) did not result in shifts in the low q-range indicating no interparticle interference or other concentration dependent effects occurred during data collection. For the product bound scatter curves of nADLS and hADSL, a final concentration of 1 mM product(s) was added to the sample. To confirm the difference seen between the scatter curves of AMP/fumarate bound nADSL and hADSL (and increase the signal/noise ratio), SAXS data collection on these samples was repeated in triplicate, collecting each time 15 frames on a sample of 50 µL. The data of all collected SAXS curves was of sufficient quality for further data analysis as determined by Guinier analysis and the pairwise distribution function calculated using the PRIMUS and GNOME programs available within the ATSAS software package^53–55^. The calculation and fitting of the theoretical SAXS curve from the different crystal structures with the experimentally collected SAXS curves (between a q-range of 0.07 nm^−1^ and 4 nm^−1^) was performed by the CRYSOL program and included a constant subtraction to remove systematic errors^56^. The obtained Chi2 values were 9.4 for the fit of the calculated SAXS curve from the apo nADSL structure with the experimental SAXS curve of apo nADSL, and 3.2 for the fit of the calculated SAXS curve from tetramer EFGH of the AICAR/fumarate bound nADSL structure with its experimental SAXS curve. For comparison, the fit of the calculated SAXS curve from the apo nADSL structure with the experimental AICAR/fumarate nADSL curve yielded a Chi^2^ value of 44.9.

### Enzymatic activity assay

The enzymatic assay was performed as described in Ray *et al*.^12^. In short, the conversion of SAMP into AMP and fumarate was monitored at room temperature by following the decrease in absorbance at λ = 282 nm in a 500 µL quartz cuvette on a UV-2401PC UV-Vis spectrophotometer (Shimadzu). The experiment was repeated in triplicate for each ADSL variant. The final protein concentration in the cuvette was 0.01 mg/mL for all ADSL variants except for the hADSL R396C mutant where a concentration of 0.02 mg/mL was used. The SAMP concentrations were varied between 11.25 and 0.35 µM. Each reaction was followed for 60 seconds and the difference in extinction coefficient (of 10,000 M^−1^ cm^−1^) between SAMP and AMP at λ = 282 nm was used to calculate the specific ADSL activity at each SAMP concentration. Kinetic parameters of the forward reaction were calculated by fitting the initial velocity data to a cooperative enzymatic model (v = V_max_* S^h^/(K_0.5_^h^ + S^h^)) in which v is the initial catalytic velocity, V_max_ is the maximal catalytic velocity, S is the substrate (SAMP) concentration, K_0.5_ is the substrate concentration yielding half the V_max_ and h the Hill coefficient. The overall set up for the assay monitoring the reverse enzymatic reaction was identical as for the forward reaction with the exception that 1 mM AMP was added to the protein solution prior to performing the assay. The reverse enzymatic reaction was followed by varying the fumarate concentration between 375 and 6.6 µM and the kinetic parameters were calculated by fitting the initial velocity data to the Michaelis-Menten equation.

### Isothermal titration calorimetry

ITC experiments were performed in triplicate with a MicroCal ITC200 Instrument (GE Healthcare). The product AMP at a concentration of 1 mM (solubilised in 10 mM HEPES pH 7.5, 100 mM NaCl, 1 mM DTT) was titrated into the cell containing 90 µM hADSL or nADSL at 20 °C. For each experiment a preliminary 1 μL injection (not included in data analysis) was followed by 20 injections of 2 μL each at a stirring speed of 800 rpm and in intervals of 180 seconds. The data were fitted using MicroCal Origin version 7.0. A blank measurement in which AMP was titrated into the cell containing only buffer displayed a very low heat of dilution for AMP and no apparent binding signal (Fig. S3).

## Electronic supplementary material

Supplementary Information

## Data Availability

The described crystal structures are accessible through the Protein Data Bank with PDB code 5NX8 for apo nADSL, PDB code 5NX9 for AMP/fumarate bound nADSL and PDB code 5NXA for AICAR/fumarate bound nADSL. The SAXS data is deposited in the Small Angle Scattering Biological Data Bank (SASBDB) under the project name ‘Human and Neanderthal Adenylosuccinate lyase’. All other data are available on request from the authors.
