# Supplementary material for: Molecular comparison of Neanderthal and Modern Human adenylosuccinate lyase
Source: Sci Rep. 2018 Dec 20;8:18008. doi: 10.1038/s41598-018-36195-5 (PMC6301967; doi:10.1038/s41598-018-36195-5)
Supplement: Supplementary file 1 — Supplementary Information [file 41598_2018_36195_MOESM1_ESM.pdf]

Supplementary Information for

Molecular comparison of Neanderthal and Modern Human adenylosuccinate lyase

Bart Van Laer[1], Ulrike Kapp[1], Montserrat Soler-Lopez[1], Kaja Moczulska[2,3], Svante Pääbo[2], Gordon Leonard[1] and Christoph Mueller-Dieckmann[1*]

[1] Structural Biology Group, European Synchrotron Radiation Facility, CS 40220, F-38043 Grenoble, France.

[2] Max Planck Institute for Evolutionary Anthropology, D-04103 Leipzig, Germany.

[3] Present address: The Francis Crick Institute, London NW1 1AT, United Kingdom.

* Correspondence: christoph.mueller_dieckmann@esrf.fr

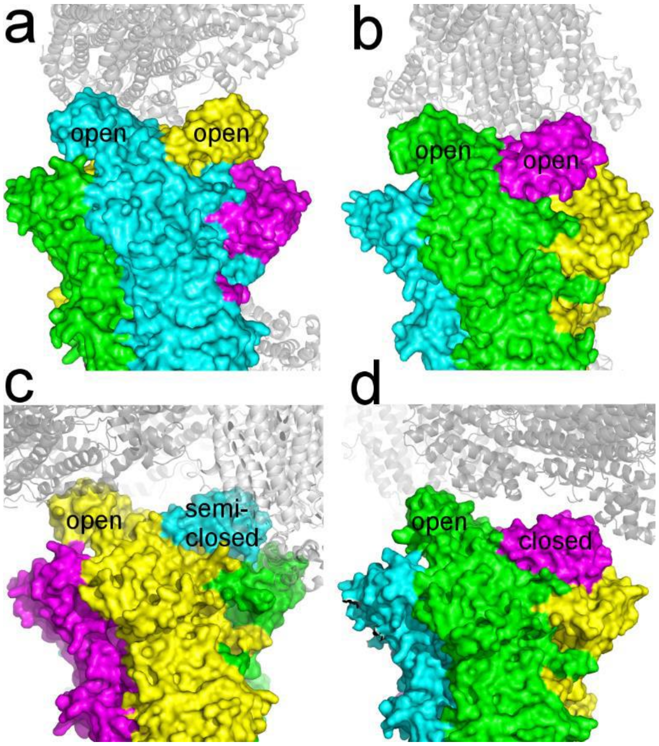

Figure S1. Comparison of crystal contacts in the AMP/fumarate (a) and AICAR/fumarate (b) bound nADSL structures. Similar to the hADSL structure, a symmetry mate (in grey cartoon representation) bridges the two domains 3 locking them it in the open conformation. These crystal contacts are not present in the AICAR/fumarate bound nADSL structure with domain 3 in the semi-closed (c) and closed (d) conformation.

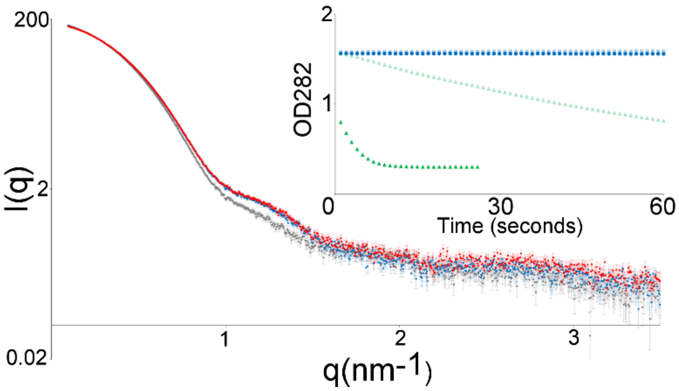

Figure S2. Superposition of the SAXS curves of an inactive nADSL His159Asn mutant in its apo (grey), AMP/ fumarate bound (blue) and SAMP bound (red) state. The inlet shows the time dependent conversion of 14 µM SAMP by nADSL (dark green triangles: assay at a protein concentration at 0.1 mg/mL, light green triangles: assay at a protein concentration of 0.01 mg/mL) and the nADSL His159Asn mutant (dark and light blue circles at the same protein concentrations as native nADSL), confirming that the nADSL His159Asn mutant is catalytically inactive.

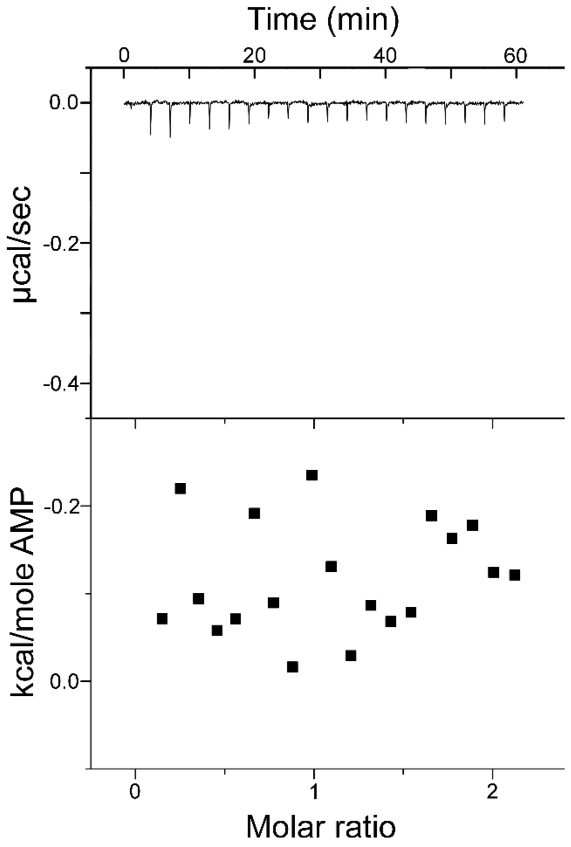

Figure S3. Control experiment for the isothermal titration calorimetry assay showing a very low heat signal upon injecting AMP into buffer and displays no apparent binding signal.

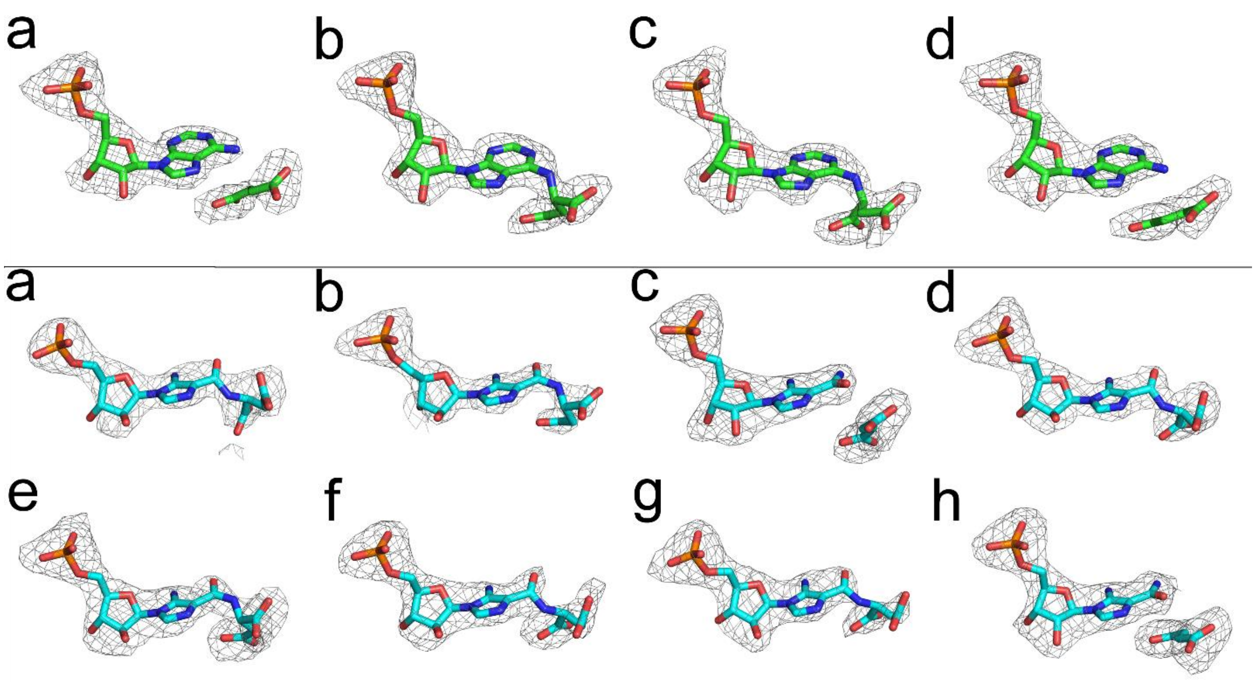

Figure S4. Omit maps (F_0-F_c) contoured at 3σ level of the ligands present in the active sites of the AMP/fumarate bound (top panel) and AICAR/fumarate bound (bottom panel) nADSL crystal structure. In the AMP/fumarate bound structure two of the four active sites (chains b and c) contain the substrate SAMP instead of the products AMP and fumarate. Similarly, in the active sites of the AICAR/fumarate bound crystals structure six of the eight active sites (chains a, b d, e, f and g) contain the substrate SAICAR.

	Apo nADSL	AMP/fumarate bound nADSL	AICAR/fumarate bound nADSL
**Data collection and processing**			
X-ray source	ESRF ID29	ESRF ID30A-3	ESRF ID30A-3
Wavelength (Å)	0.976	0.967	0.967
Resolution (Å)	48 – 1.7 (1.8 - 1.7)	48 – 2.3 (2.4 – 2.3)	49 – 2.4 (2.5 – 2.4)
Total/Unique reflections	975,395 / 211,102 (144,594 – 30,633)	328,477 / 86,413 (48,950 / 12,466)	694,849 / 153,910 (104,095 / 22,475)
Space group	$P2_12_12_1$	$P2_12_12_1$	C2
Cell dimensions			
a, b, c (Å)	85.4, 104.9, 215.4	85.7, 107.5, 209.7	356.5, 74.2, 160.3
α, β, γ (°)	90.0, 90.0, 90.0	90.0, 90.0, 90.0	90.0, 108.9, 90.0
R_{meas} (%)	11.6 (130.3)	23.5 (138.9)	22.1 (139.6)
$\langle I/\sigma(I) \rangle$	9.8 (1.2)	5.7 (1.2)	6.8 (1.2)
$CC_{1/2}$ (%)	99.8 (42.4)	98.6 (35.6)	99.0 (45.1)
Completeness (%)	99.5 (99.8)	99.6 (99.8)	99.0 (99.9)
Redundancy	4.6 (4.7)	3.8 (3.9)	4.5 (4.6)
**Model Refinement**			
Protein molecules / ASU	4	4	8
R_{work}/R_{free} (%)	17.1 / 21.5	19.5 / 22.8	22.1 / 25.4
RMSD bond length (Å)	0.015	0.011	0.011
RMSD bond angle (°)	1.49	1.33	1.36
Ramachandran favoured/allowed/disallowed (%)	98.3 / 1.5 / 0.2	99.1 / 0.6 / 0.3	97.9 / 1.8 / 0.3
PDB code	5NX8	5NX9	5NXA

Table S1. X-ray diffraction data collection, refinement and validation statistics. Values for the highest resolution shell are given in brackets.

**Data-collection parameters**					
Instrument	ESRF BM29				
Wavelength (Å)	0.99				
q-range ($Å^{-1}$)	0.0032 – 0.49				
Sample-to-detector distance	2.864 m				
Exposure time (sec)	1 per frame				
Concentration range	1.25 – 5 mg/ml				
Temperature (K)	293				
Detector	Pilatus 1M (Dectris)				
Flux (photons/s)	1 × 1012				
Beam size (μm^2)	700 × 700				
**Structural parameters for hADSL**	**Apo**	**AMP bound**	**AMP/fumarate bound**	**AICAR bound**	**AICAR/Fumarate bound**
I_0 (cm^{-1}) [from Guinier]	0.0154	0.0153	0.0152	0.0153	0.0152
R_g (nm) [from Guinier]	3.72	3.69	3.62	3.69	3.62
$q_{min}R_g – q_{max}R_g$ used for Guinier	0.17 - 1.29	0.26 - 1.28	0.17 – 1.29	0.28 – 1.29	0.17 – 1.29
D_{max} (Å) [from p(r)]	105	105	102	104	102
q-range used for p(r) ($Å^{-1}$)	0.05 – 0.38	0.07 – 0.38	0.05 – 0.38	0.08 – 0.38	0.05 - 0.38
Porod volume V_p ($Å^3$) [from Scatter]	291×10^3	320×10^3	286×10^3	280×10^3	273×10^3
Molecular mass M_r (kDa) [from V_p]	171	188	168	165	161
Calculated tetrameric M_r from sequence (kDa)	220	220	220	220	220
**Structural parameters for nADSL**	**Apo**	**AMP bound**	**AMP + Fumarate bound**	**AICAR bound**	**AICAR + Fumarate bound**
I_0 (cm^{-1}) [from Guinier]	0.0154	0.0154	0.0153	0.0154	0.0152
R_g (nm) [from Guinier]	3.70	3.71	3.65	3.71	3.62
$q_{min}R_g – q_{max}R_g$ used for Guinier	0.21 – 1.29	0.29 – 1.29	0.20 – 1.29	0.29 – 1.29	0.19 – 1.29
D_{max} (Å) [from p(r)]	105	105	104	106	101
q-range used for p(r) ($Å^{-1}$)	0.07 – 0.38	0.08 – 0.38	0.06 – 0.38	0.08 – 0.38	0.01 – 0.38
Porod volume V_p ($Å^3$) [from Scatter]	315×10^3	301×10^3	288×10^3	282×10^3	277×10^3
Molecular mass M_r (kDa) [from V_p]	185	177	169	166	163
Calculated tetrameric M_r from sequence (kDa)	220	220	220	220	220

Table S2. SAXS data collection and validation statistics.

	Forward reaction (SAMP)		Reverse reaction (Fumarate)	
	**hADSL**	**nADSL**	**hADSL**	**nADSL**
V_{max} (µmol converted / min / mg ADSL)	11.5 ± 0.5	11.2 ± 0.5	2.3 ± 0.1	2.4 ± 0.1
Hill coefficient	1.6 ± 0.3	1.8 ± 0.3	NA	NA
$K_{0.5}$ or K_m (µM)	1.1 ± 0.1	1.1 ± 0.1	79.7 ± 8.0	66.5 ± 7.2

Table S3. Kinetic parameters of hADSL and nADSL at 20°C.

	**hADSL**			**nADSL**		
	run 1	run 2	run 3	run 1	run 2	run 3
Number of AMP binding sites / protein molecule (n)	0.9	1.0	1.1	1.1	1.1	1.1
K_D (µM)	23.2	25.1	21.0	29.1	23.6	27.1
ΔS (cal/mol/°C)	-13.0	-12.4	-7.1	-15.9	-12.7	-12.9

Table S4. Binding parameters of nADSL and hADSL for AMP as determined by isothermal titration calorimetry.

	**hADSL**	**hADSL R396H**	**hADSL D422Y**	**hADSL R426H**
V_{max} (µmol SAMP converted / min / mg ADSL)	12.1 ± 0.4	1.5 ± 0.1	4.2 ± 0.1	9.3 ± 0.3
Hill coefficient	1.6 ± 0.2	NA	1.9 ± 0.3	1.4 ± 0.2
$K_{0.5}$ or K_m (µM)	1.0 ± 0.1	0.19 ± 0.02	0.36 ± 0.03	0.75 ± 0.06

Table S5. Kinetic parameters for the forward reaction of native hADSL and ADSL deficiency-causing hADSL mutants at 20°C.
